# Effectiveness of interprofessional oral health program for pediatric nurse practitioner students at Northeastern University, United States

**DOI:** 10.1186/s12903-019-0861-y

**Published:** 2019-08-01

**Authors:** Azita Khanbodaghi, Zuhair S. Natto, Martha Forero, Cheen Y. Loo

**Affiliations:** 1 Department of Pediatric Dentistry, Tufts University School of Dental Medicine, 1 Kneeland Street, Boston, MA 02111 USA; 2 Department of Dental Public Health, Faculty of Dentistry, King Abdulaziz University, Jeddah, Saudi Arabia

**Keywords:** Knowledge, Awareness, Pediatric nurse practitioner students, Oral health, Interprofessional education, Interdisciplinary communication

## Abstract

**Background:**

Interprofessional education (IPE) is an important part of the landscape of modern education. However, there is a significant deficiency of studies that evaluate IPE in dentistry. The aim of this article is to evaluate the effects of an oral health educational program on the dental knowledge, awareness, attitude, confidence, and behavior of pediatric nurse practitioner (PNP) students and to emphasize the importance of IPE for PNP.

**Methods:**

First-year pediatric nurse practitioners from Northeastern University participated in an IPE oral health education seminal and practical session as a pilot study. Several tests were used to evaluate the effectiveness of the educational program. The post-test assessed the knowledge, awareness, attitude, confidence, and behavior of the students immediately after attending the lecture; again immediately after hands on experience; and finally at a follow-up approximately a month after attending the training module. The training module consists of prevention and anticipatory guidance; caries process and management; trauma and dental emergencies. Differences in score items were evaluated between 4 time points. Friedman’s, Wilcoxon signed-rank and McNemar’s tests were used to analyze the results.

**Results:**

Knowledge score was determined based on the number of correct responses to seven questions, while awareness score was based on the median of eight questions. Fifteen confidence, attitude, and behavior questions were used. The total sample size was 16 students with a mean age 33.31 ± 7.52. The majority were females (87.5%). Significant improvement was seen in all subjects’ overall knowledge of oral health topics. The confidence, attitude, and behavior scores were differed by time of test (*P* < 0.01). It was the highest after post-test and the lowest in pre-test.

**Conclusion:**

Our study suggests that introducing an Interprofessional education program for pediatric nurse practitioner students can provide them with adequate knowledge, awareness, confidence, and attitude regarding oral health issues. It also can help them in changing behavior, prevention and ongoing dental surveillance.

## Introduction

Interprofessional education (IPE) is a hotly debated area of education. It means all students from different professional training groups come together for a collaborative learning practice and as members of an interdisciplinary team focusing on client- or patient-centered health care [1, 2]. There are several organizations who vocally support IPE practice, such as The World Health Organization (WHO), the Institute of Medicine (IOM), and the American Public Health Association (APHA) [2–6]. It has several advantages, including decreases in patients’ cost and length of stay, improvements in the quality of patient care, and reduces medical errors [2, 7].

Early childhood caries (ECC) is increasing among 2–5-year-olds, from 24 to 28% based on a National Health and Nutrition Examination Survey (NHANES), and is one of the most prevalent diseases in pediatric patients. There are several options that may help to reduce this disease, such as newborn nursery nurses and pediatric nurses. Both nurses have an exclusive opportunity to positively influence the current situation in children by providing comprehensive oral health education and guidance for new parents. Thus, these nurses are in an ideal position to start educating parents about oral health care [8]. In the dental field, one study recommends providing stronger interdisciplinary oral health educational programs for non-dental providers (pediatric nurse practitioner students). It is suggested that this solution would improve all educational aspects, such as knowledge, attitude, and behavior [9]. Introducing a program will help in fluoride varnish applications, oral-systemic health relationship, and the dental home care, which will help in reducing the incidence of ECC as a sequence [8].

Although the mouth has specific features that create a unique element in the body, the majority of professionals underestimate the oral cavity’s association with human health and often mistakenly separate mouth issues from the rest of the body [10]. IPE gives all residents the opportunity to reestablish the connection between systemic or general health and oral health, and to overcome the profession’s previous separation from medical care and its recompensation programs. An advanced practice for nurses is crucial as well, which can lead to better prevention in several dental aspects, such as dental caries.

The health needs for any patient, in most cases, requires more than one discipline to address different health concerns in a disease [11, 12]. Oral health is a discipline that is especially related to several systemic diseases. Introducing the didactic educational component of dental professional in interdisciplinary exposure will be beneficial though the implementation of IPE experiences [13]. In particular, early interactions between different students during IPE could help them to understand the overall issues and the importance of interpersonal skills in the healthcare workplace [2, 7, 14]. This will address the concern of low knowledge among physicians regarding oral health [15], the relationship between periodontal disease and systemic health [16, 17], Bisphosphonate-related osteonecrosis of the jaw (BRONJ) [18], and oral cancer [19, 20].

Nurses represent the largest group in the health field in the United States and provide the initial contact with patients in many cases, putting them in a position where they can significantly help reduce oral health issues. As a result of the increase in chronic disease and elderly populations, disparities and care cost, the need of high-quality primary care services will increase and the number of primary care physicians and professionals will decrease. In these situations, pediatric nurse practitioners (PNP) will have a great value as the solution to this crisis [21]. However, there is deficiency in the studies that evaluate IPE among nurses overall in dentistry. For this reason, the aim of this article is to describe the importance and the effect of interprofessional education (IPE) in oral health education, and how it will help pediatric dentistry and mainly nurse practitioners in improve their dental knowledge, awareness, attitude, confidence, and behavior with this approach.

## Materials and methods

This is a pilot study and it was approved by the institutional Review Board for The Behavioral and Health Sciences at Tufts School of Dental Medicine (#12088). Sixteen first-year PNPs from Northeastern University participated in an IPE and oral health education seminal and practical session during spring term of 2016.

### Data collection

Several surveys were used to evaluate the effectiveness of the educational program. Tests were implemented by administering a self-reported survey to assess the changes in students’ knowledge before and after the training module. This survey is similar to that administered by Czarnecki, et al. (2014) to assess whether an IPE would improve nursing students’, dental students’, and pediatric dentistry residents’ knowledge of children’s oral health between the beginning and the end of the experience. Each survey consisted of seven knowledge-based questions - four confidence and three attitude. In addition, awareness and behavior was covered (eight each). Responses were ranged from 1 (lowest: very unlikely, strongly disagree, not confident at all) to 5 (the highest, which were the opposite). The knowledge section was multiple choice questions. The survey was validated in the original article and was not modified in current population sample.

The pre-test assessed the knowledge, awareness, attitude, confidence, and behavior of pediatric nurse practitioner students regarding oral health issues/practice at baseline, immediately prior to attending the training module. The post-test assessed the same components (a) immediately after attending the lecture; (b) immediately after hands-on and (c) at a follow-up approximately one month after attending the training module. The schedule is outlined in Table 1.

**Table 1:** The component of oral Health Educational Program for Pediatric Nurse Practitioner

	*Components of Training Module*	*Time*
Introduction and pre Assessment of knowledge	• Introduction and rationale of study • Distribute pre-test to students	15 min
Didactic Component	• Power point presentation for educational intervention -Prevention and anticipatory guidance -Caries process and management -Trauma and dental emergencies • Question and answer session	1 h
Assessment of knowledge	• Distribute first post-test to students	15 min
Hands- On	• Consists of using manikin, typodont and videos learning about: -Fluoride application -Nutrition consultation -Caries risk assessment -Knee to knee examination	1 h
Assessment of knowledge	• Distribute second post-test to students	15 min
Assessment of knowledge	• Third post-test (approximately one month follow up) by email	One month follow up

When the students arrived on the day of seminar, they received the pre-test survey with a randomly assigned number for each subject and the subjects provided email addresses, which linked to that number. All the information was collected by an impartial investigator and was followed by an overview of the module. Next, a one hour PowerPoint presentation was given, which included prevention and anticipatory guidance, caries process and management, trauma, and dental emergencies, followed by a question–and-answer session and first post-test. Next all the subjects were randomly divided in groups of two and participated in a practical session consisting of fluoride application and caries assessment risk. The second post-test was distributed after the session. All participants who completed the pre-test, first post- test, and second post-test were given a gift card to Starbucks Coffee Company. A one month follow-up email was sent, and all the participants who responded to the email then received an additional gift card.

### Sample size

All 16 students in the program were recruited as participants to allow for possible dropout. A power calculation was conducted using G*power program (version 3.1.9). Based on previously unpublished collected data (Effectiveness of Oral Health Education for Pediatric Nurse, J. Kyle Stark) in the same department and a rate of 3.6% for the percentage correct decreased from post-test to “post-post-test” several months later. A sample size of 16 participants would result in a Type I error rate of 5% and a power greater than 90%.

### Statistical analysis

Questions regarding differences in the attitude, behavior, and confidence questions were evaluated using Friedman’s test to compare the four time points (pre-test, post-test, the “post-post-test,” and one month later) and Wilcoxon signed-rank test along with Bonferroni correction or McNemar’s test. Internal consistency was assessed using Cronbach’s alpha intraclass correlation (ICC). All analyses were performed using SAS Version 9.3 (SAS Institute, Cary, NC). Any *p*-value less than 0.05 was considered statistically significant.

## Results

A total of 16 students were included in the study. All students completed the complete training program and the four surveys. The mean age was 33.31 ± 7.52. The majority were females (87.5%). Internal consistency (intraclass correlation, ICC) for each group (knowledge and awareness) was explored using Cronbach’s alpha and it was above 0.60. These scores were differed by time of test (*P* < 0.01). It was the highest after post-post-test and the lowest in pre-test.

### Oral health knowledge

**Fig. 1:**
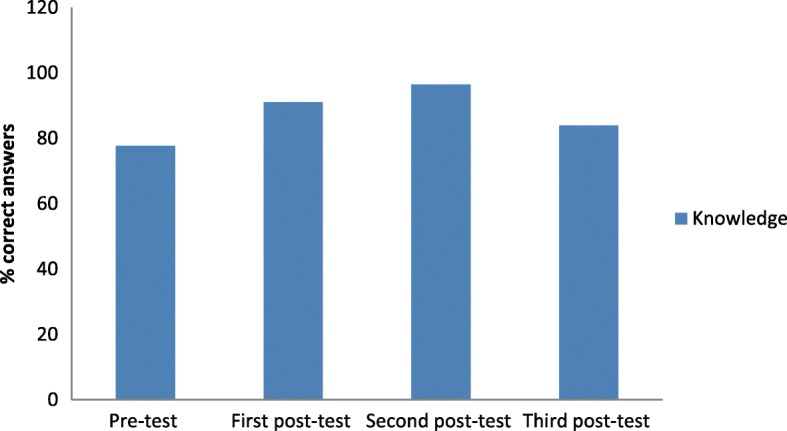
proportion of students got the correct answers in overall knowledge

Measures of the participants’ oral health knowledge during the program are shown in Table 2. The knowledge score was determined based on the number of correct responses to seven questions. After the program, a significant improvement was seen in all subjects’ overall knowledge of oral health topics in post-test, post-post-test, and one month (sum of number of student got the correct answer (percentage) =14.57(91.07), 15.43(96.43), and 13.43(83.93), respectively, freedman test, *p* < 0.001) (Fig. 1).

**Table 2:** number (percentage) of correct answers in knowledge section

Question	Pre test *N* = 16	Post test N = 16	Post post test N = 16	1 month *N* = 16	*P* value
Children first dental visit	12(75)	16(100)	16(100)	16(100)	.3916
Caries location and brushing	11(68.75)	11(68.75)	14(87.50)	8(50)	.0645
Bacteria causing tooth decay	14(87.50)	16(100)	16(100)	16(100)	.1116
Age of referral	12(75)	16(100)	16(100)	16(100)	.3916
Risk factor for oral trauma	12(75)	12(75)	14(87.50)	10(62.50)	.2436
Caries risk factors	10(62.50)	15(93.75)	16(100)	12(75)	.0122*
Population group risk	16(100)	16(100)	16(100)	16(100)	1.00
Overall	12.43(77.68)	14.57(91.07)	15.43(96.43)	13.43(83.93)	.0002*

*p < 0.05

In post-test, five questions were answered correctly by more than 85% of the participants. It goes to 7 questions in post-post-test. However, it came back to five correct questions again after a month. Specifically, 100% of the participants answered the questions about children’s first dental visit, bacteria causing tooth decay, age of referral, and population group risk correctly in all time points in post assessment. Caries risk factors question was the only significant answer in all time points (*p* = 0.0122).

### Oral health awareness

**Fig. 2:**
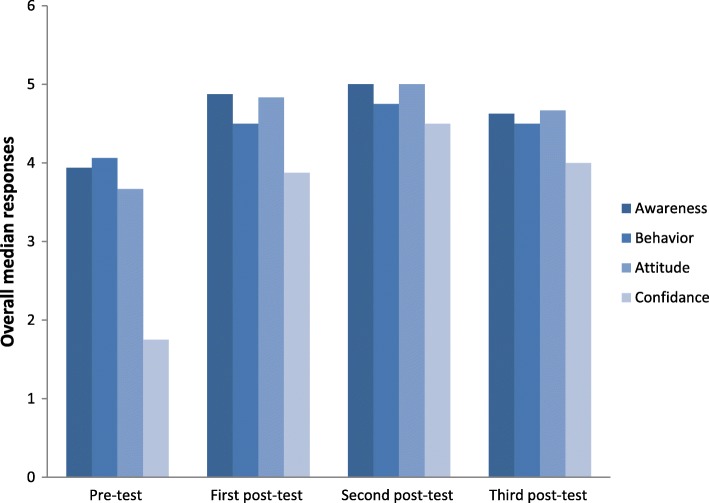
Median of students in overall awareness, behavior, attitude and confidence

Measures of the participants’ oral health awareness are shown in Table 3. There was a significant improvement in all awareness items, such as awareness about bottle use, oral habits, dental counsel, teeth examination, fluoride intake and so on, during and after the program. The overall awareness score was determined based on the median of eight questions. After the program, a significant improvement was seen in all subjects’ overall awareness (Fig. 2).

**Table 3:** Median (interquartile range) of each question in awareness section

Question	Pre test N = 16	Post test *N* = 16	Post post test N = 16	1 month *N* = 16	*P* value
Inquire about bottle use	4.5(4.0–5.0)	5.0(4.5–5.0)	5.0(5.0–5.0)	5.0(5.0–5.0)	.0046*
Inquire about oral habits	4.5(4.0–5.0)	5.0(5.0–5.0)	5.0(5.0–5.0)	5.0(4.5–5.0)	0.0074*
Examine a child’s teeth	3.0(2.0–4.0)	5.0(4.0–5.0)	5.0(5.0–5.0)	5.0(3.5–5.0)	<.0001*
Counsel the dentist	4.0(4.0–5.0)	5.0(4.5–5.0)	5.0(5.0–5.0)	5.0(5.0–5.0)	<.0001*
Counsel on tooth brushing	4.5(4.0–5.0)	5.0(5.0–5.0)	5.0(5.0–5.0)	5.0(4.5–5.0)	.0110*
Assess fluoride intake	4.0(3.0–5.0)	5.0(4.0–5.0)	5.0(5.0–5.0)	5.0(4.0–5.0)	.0011*
Inquire about mothers’ dental health	3.0(2.0–3.0)	5.0(4.0–5.0)	5.0(5.0–5.0)	4.0(3.0–4.5)	<.0001*
dietary counseling	4.0(3.0–4.5)	5.0(4.5–5.0)	5.0(5.0–5.0)	5.0(4.0–5.0)	<.0001*
Overall	3.93(3.43–4.25)	4.88(4.38–5.0)	5.0(5.0–5.0)	4.63(4.19–4.94)	<.0001*

*p < 0.05

### Oral health confidence

Confidence scores during the program are shown in Table 4. There was significantly more confidence in their ability to identify teeth with cavities, plaque, enamel demineralization, and in their ability to apply fluoride varnish both during and after the program. Median score was determined based on the median of all confidence questions. After the program, a significant change was noticed in overall confidence score (Fig. 2).

**Table 4:** Median (interquartile range) in confidence section

Question	Pre test N = 16	Post test *N* = 16	Post post test N = 16	1 month N = 16	*P* value
Ability to identify teeth with cavities	1.5(1.0–3.0)	4.0(2.5–4.0)	4.5(3.5–5.0)	4.0(3.0–4.0)	<.0001*
Ability to Identify teeth with plaque	2.0(1.5–3.0)	4.0(3.5–5.0)	4.5(4.0–5.0)	4.0(3.5–4.0)	<.0001*
Ability to Identify enamel demineralization	1.5(1.0–3.0)	4.0(3.0–4.5)	4.5(3.5–5.0)	4.0(3.0–4.0)	<.0001*
Ability to apply fluoride varnish	1.0(1.0–2.5)	4.0(3.0–5.0)	5.0(5.0–5.0)	4.0(4.0–5.0)	<.0001*
Overall	1.75(1.13–2.88)	3.88(2.88–4.5)	4.5(4.0–5.0)	4.0(3.25–4.25)	<.0001*

*p < 0.05

### Oral health attitude

Oral health attitude was calculated in the same way and it showed the same pattern after the program (Table 5 and Fig. 2). There was more agreement for the items including oral examination and risk assessment, counseling on prevention, and fluoride application, as an important part of routine child care.

**Table 5:** Median (interquartile range) in attitude section

Question	Pre test N = 16	Post test N = 16	Post post test N = 16	1 month N = 16	*P* value
Oral examination and risk assessment for dental problems during physical examinations	4.0(4.0–5.0)	5.0(5.0–5.0)	5.0(5.0–5.0)	5.0(4.5–5.0)	<.0001*
Counseling on prevention of dental problems	4.0(4.0–5.0)	5.0(5.0–5.0)	5.0(5.0–5.0)	5.0(5.0–5.0)	<.0001*
Application of fluoride varnish	3.0(2.0–4.0)	4.5(4.0–5.0)	5.0(5.0–5.0)	4.0(3.0–5.0)	<.0001*
Overall	3.67(3.33–4.0)	4.83(4.5–5.0)	5.0(5.0–5.0)	4.67(4.17–5.0)	<.0001*

*p < 0.05

### Oral health behavior

Participants’ responses regarding oral health behavior are shown in Table 6 and calculated in the same way. Although an overall improvement was documented (Table 6 and Fig. 2), some specific items did not show any changes, such as referrals for dental trauma without a fracture, mouth guard recommendation, and referrals for type II, III, and IV fracture.

**Table 6:** Median (interquartile range) in behavior section

Question	Pre test N = 16	Post test N = 16	Post post test N = 16	1 month N = 16	*P* value
Tooth brushing instruction	2.5(1.0–3.0)	3.0(3.0–3.0)	3.0(3.0–3.0)	3.0(2.5–3.0)	.0193*
Refer for dental trauma without a fracture	4.5(4.0–5.0)	5.0(4.0–5.0)	5.0(4.0–5.0)	5.0(4.0–5.0)	.5222
Refer for Type I fracture	4.0(4.0–5.0)	5.0(4.0–5.0)	5.0(4.5–5.0)	5.0(4.0–5.0)	.0468*
Refer for Type II fracture	5.0(4.0–5.0)	5.0(4.0–5.0)	5.0(5.0–5.0)	5.0(5.0–5.0)	.0798
Refer for Type III fracture	5.0(5.0–5.0)	5.0(5.0–5.0)	5.0(5.0–5.0)	5.0(5.0–5.0)	.4684
Refer for Type IV fracture	5.0(5.0–5.0)	5.0(5.0–5.0)	5.0(5.0–5.0)	5.0(5.0–5.0)	.4684
Mouth guards recommendation	5.0(5.0–5.0)	5.0(5.0–5.0)	5.0(5.0–5.0)	5.0(5.0–5.0)	.3916
Management of an avulsed tooth	2.0(1.0–3.0)	4.0(4.0–5.0)	5.0(4.5–5.0)	4.0(3.5–5.0)	<.0001*
Overall	4.06(3.88–4.25)	4.5(4.25–4.63)	4.75(4.5–4.75)	4.5(4.31–4.63)	<.0001*

*p < 0.05

## Discussion

Our results indicated that knowledge, awareness, attitude, confidence, and behavior of the pediatric nurse practitioner students were all improved through engagement in the IPE program. Previous articles suggested that a traditional didactic education did not improve physician performance. Hence, delivering effective education using IPE during residency training has been successful and less challenging as we did [22–24].

The pediatric primary care nurse practitioner program was an excellent target in pediatric dentistry. It is designed to prepare nurses in the primary healthcare pediatric population with the required knowledge and skills. The core curriculum contained a variety of topics in community health program, private and public day care, clinics, schools and home programs, outpatient, and chronic care facilities [25]. Our program provided an enhanced method to the current educational program to support IPE concept.

It is important for the nurse practitioners to have adequate knowledge about oral health due to another important factor. Currently, 40 % of hospitalized children required oral healthcare needs [26]. It can complicate treatment or can happen as a consequence of medical conditions or treatments [26]. As a result, minimal attention will be provided to oral hygiene compared with other medical issues [26]. Therefore, nurses can play an important role in these cases and they can act as a bridge between patients, parents, physicians, and dentists.

Moreover, there are several examples of how IPE can help these nurses. In cancer patients, for example, patients have difficulty speaking, swallowing, eating, and infection. Nurses can benefit from IPE regarding oral care in chemotherapy and/or radiotherapy [27]. Also, the provision of effective oral care to intubated patients is a complex issue. Through IPE, this issue can be tackled by motivated, educated critical care nurses who have within their grasp the power to make significant improvements to the care of their patients [28].

The oral health care of pregnant women is another area that will greatly benefit from IPE. Nurses are one of the main providers of antenatal healthcare services and play important roles in increasing the awareness of oral health and dissemination of information on oral health care to pregnant women [29].

Knowledge is an important factor for any IPE. This can be acquired by didactic education, simulation exercises, and clinical observation. However, it is maybe insufficient for dental referrals and it needs additional methods to gain providers’ confidence [26]. Medical education should include the practice of oral health risk assessment, early detection, and dental referral services [26]. In addition, it would help in reducing the cost of treatment, dental education, and increasing health case access. IPE model is building bridges among the health professions in basic, social, and clinical science education, practicing, research, and community service specifically between nurses and dentists [30]. The results of a study found a 38% partial or total overlap of the core competencies between dental and nursing professions [30]. However, it may be difficult in some situations. For this reason, multidisciplinary approach (MDA) is just one initial step that will help in applying IPE.

Moreover, interprofessional education is very important in the referral system. Health professionals, especially primary care practitioners who able to screen patients for dental disease, were more likely to refer their patients if they have any issue orally compared to those who lacked confidence [31]. In a study, a primary care pediatric practitioners was able to identify dental decay among children with good specificity (92–100%) and sensitivity (87–99%) after two hours of oral health training program [32, 33]. These results recommend incorporating dental screening in a primary care pediatric clinic, which could improve the oral health of children and parent as well [32, 33]. This behavior seems to be promising and beneficial. In our study, some behavior items did not change, possibly because of previously existing behavior practice of referring pediatric patients and adequate education program regarding specific items.

Although IPE is facing many challenges such as funding, program willingness, leadership interest, faculty/practitioner attitude, and interest, it would have great value in pediatric dentistry. In fact, the AAP policy statement of 2003 recommended several basic preventive strategies that can help to reduce dental caries among pediatric population. One of them is the engagement of pediatricians in oral health assessment of both parents and children. In addition, this would increase their awareness and attention to the early childhood dental caries. The pediatric primary care nurse practitioner will be an excellent target as well to have the required knowledge and skill in the primary healthcare pediatric population. This will provide an excellent bridge between patients, parents, physicians, and dentists. In addition, it will help in decision-making regarding timely and effective intervention.

IPE may provide nurse practitioners and other non-dental healthcare providers the adequate motivation, confidence, and attitude regarding oral health issues. This will help any specialty, including pediatric, toward oral health consultations/screening, prevention, and referrals. In addition, it will improve oral health care to young children using established medical home.

The sample size was small in this study. However, this project was conducted for the first time. In addition, pediatric nurse practitioner students were a small group and we included all of them. Another limitation is the short follow up. However, a longer follow-up study will be conducted in the future.

## Conclusion

Our study suggests that introducing an Interprofessional education program for pediatric nurse practitioner students can provide them with adequate knowledge, awareness, confidence, and attitude regarding oral health issues. It can help them in changing behavior, prevention and ongoing dental surveillance. The program is raising the importance of oral health components in nursing programs and potentiality non-dental professionals, and in a referral system between pediatric nurse practitioners with pediatric/general dentists.

## Data Availability

The dataset used during the study are available from the corresponding author upon request.

## Abbreviations

APHA: The American Public Health Association
BRONJ: Bisphosphonate-related osteonecrosis of the jaw
ECC: Early childhood caries
IOM: The Institute of Medicine
IPE: Interprofessional education
NHANES: National Health and Nutrition Examination Survey
PNP: pediatric nurse practitioner
WHO: The World Health Organization
